# Supplementary material for: Are carbon emissions trading and green financial instruments synergistic? -Comprehensive quantitative research based on content analysis
Source: PLoS One. 2024 Mar 7;19(3):e0298601. doi: 10.1371/journal.pone.0298601 (PMC10919601; doi:10.1371/journal.pone.0298601)
Supplement: S1 Data — (ZIP) [file pone.0298601.s001.zip › Data/地级市Z_F工作报告.zip!/╔╜╢1⁄2/╝├─╧╩╨/└┤╫╘╣1⁄2╓┌║┼í╛┬φ┐╦╩2╛▌═°í┐_2018.docx]

各位代表：

　　现在，我代表市人民政府向大会报告工作，请予审议，并请各位政协委员和其他列席人员提出意见。

一、2017年工作回顾

　　过去的一年，是济南发展史上极不平凡的一年，也是凝心聚力、攻坚克难的一年。全市上下高举习近平新时代中国特色社会主义思想伟大旗帜，在省委省政府和市委坚强领导下，紧紧围绕“打造四个中心，建设现代泉城”的中心任务，全力推进“453”工作体系，雷厉风行抓落实，快干实干促发展，圆满完成了市第十六届人大一次会议确定的各项任务，全市呈现出“经济快速发展、社会安定和谐、人民安居乐业、干部干事创业”的良好局面。

　　（一）扎实推进“四个中心”建设，综合实力明显提升。大力实施开放、融合、聚焦三大战略，省会现代化建设取得积极进展。经济竞争力持续增强。预计全市生产总值突破7000亿元，增长8%左右，增速位居副省级城市前列；固定资产投资4400亿元左右，增长13.5%左右，增幅居全省第一位；一般公共预算收入完成677.2亿元，增长10.5%，居全省第二位；税收占比达到80%，居全省第二位。主要经济指标增幅领跑全省，达到了2000年以来最好水平。金融支撑力持续增强。完成金融业增加值770亿元左右，金融机构本外币存款余额1.7万亿元，均居全省第一位；新引进中德住房储蓄银行济南分行等各类金融机构60家；中央商务区标志性项目加快推进，集聚效应逐步显现。物流影响力持续增强。社会物流总额突破2.3万亿元，增长13%，国家5A级物流企业总数达到10家，居全省第一位。中欧班列顺利开通，获评“2017中国物流业最具创新力城市”和“2017中国物流业最具影响力城市”。科技创新力持续增强。高新技术产业产值比重达到45%以上，万人有效发明专利拥有量达到25件，均居全省第一位。新技术新产品层出不穷，世界首个商用量子通信专网正式运行，国内首台氢燃料电池新能源汽车成功下线。微软全球首家金融科技孵化器、全国首个大数据流通与交易技术国家工程实验室落户我市，积蓄了发展新动能。

　　（二）积极促进产业转型升级，新旧动能转换明显加快。坚持高端高质高新方向，着力培育十大千亿级产业集群，三次产业结构调整为4.5：36.5：59。工业优化升级。规模以上工业增加值增长9.8%左右，增幅在全省位次创10年来最好水平。高端装备制造业占规模以上工业增加值比重达到55.6%。一批龙头企业带动作用凸显，重汽集团主营业务收入突破900亿元，浪潮服务器市场占有率跃居全球第三位，齐鲁制药、山东国舜入选2017中国工业品牌榜。一批中小微企业借力“互联网+”快速成长，全市上云企业数量突破3000家。现代服务业发展壮大。现代服务业占服务业比重达到55%左右，社会消费品零售总额增长10%左右，宜家、麦德龙、迪卡侬、奥特莱斯等消费新热点不断涌现；电子商务交易额、网络零售额分别增长36%和30%。举办各类展会167个，观展人数达850万人次。实现旅游消费总额964亿元、增长13.8%，服务业对经济发展的带动作用明显增强。现代农业提质增效。建成120万亩粮食高产创建示范区，电子商务村镇服务站达1529个，农业适度规模化经营率达到40%。“三品一标”达到1135家，列全省第一。设立全国首家“国家级农作物品种展示示范基地”，安信种业成为全省首个在新三板上市的种苗企业。

　　（三）加大规划建设管理力度，城乡面貌明显变化。扎实推进城市建设改造提升，城乡更加靓丽宜居。布局更科学。按照组团式发展的思路，启动济南城市发展战略、新一轮城市总体规划修编，完成土地利用总体规划调整，入选全国“城市双修”、城市设计“双试点”城市。高起点高效率推进新旧动能转换先行区、国际医学科学中心、新东站等重点片区规划建设，穿黄隧道和两座跨河大桥破土动工，“携河北跨”迈出实质性步伐。各县区城区框架逐步拉开，呈现出空间拓展、竞相发展的新格局。交通更便捷。石济客专开通运行，二环南路东延等5条快速路建成通车，轨道交通建设加快推进；开通纬十二路BRT等16条公交新线，打通了27条丁字路、瓶颈路，交通拥堵状况得到有效缓解。城区更靓丽。强力推进拆违拆临、建绿透绿，全年拆除违法建设3287万平方米，拆除违法户外广告1.7万块。整治背街小巷1600余条，拔除线杆1800多个，规范线缆2万余公里，空中“蜘蛛网”遁形入地。新增绿地230余万平方米，绿化裸露土地260余万平方米，新建一大批街景小品、口袋公园，实现了见缝插绿、一步一景。乡村更整洁。农村“七改”深入推进，完成农村危房改造3508户、无害化卫生厕所改造24.3万户、村庄街巷硬化320个、小城镇电网改造63个。省市级美丽乡村示范村总数达到179个，美丽乡村标准化覆盖率达到52%。设施更完善。完成电力线路迁改527公里，新增集中供热面积900万平方米、天然气管线185公里。新建改建旅游厕所206座，荣获“全国旅游厕所革命先进市”称号。新增市民健身场地1170处，新设便民服务网点和菜篮子直通车650余处。城市更文明。把提升品质、服务群众摆在重要位置，万众一心、众志成城，以省会城市第一名的成绩，成功摘得“全国文明城市”荣誉奖牌，实现了泉城人民20年的夙愿，市民群众的自豪感、荣誉感、归属感大大增强。

　　（四）持续强化保护治理，生态环境明显改善。牢固树立“绿水青山就是金山银山”的理念，努力打造绿色家园。天越来越蓝。深入实施大气污染防治十大措施，取缔整改“散乱污”企业7190家，全面完成323台35蒸吨及以下燃煤锅炉淘汰任务。入选国家“北方地区冬季清洁取暖试点城市”，完成气代煤、电代煤改造11万户、清洁燃煤替代42万户。对事关重大民生及环保信用优秀的工地、企业，创新实施“蓝绿名单”制度，实现了供暖季“天蓝冬暖”两不误。全年空气良好以上天数达到 185天，同比增加22天，重污染天气天数同比减少6天，久违了的“蓝天白云、繁星闪烁”，又回到了市民的身边。山越来越绿。强化山体绿化，推进“青山入城”，对170座山体划定保护红线，造林绿化5.6万亩，建成山体公园30处，为市民提供了更多亲近自然、放松身心的活动空间。水越来越清。全面落实河长制，深入贯彻“水十条”，城区37处黑臭水体治理任务全面完成；强力推动南部山区水源地违法建设整治，大力推进河道、水库、小流域治理。在去年天气干旱的情况下，采取多种措施调水补源，积极开展增雨雪作业，重点泉群连续14年保持喷涌。“听得到泉水叮咚、看得见一城山色”成为泉城靓丽的名片。

　　（五）全面深化改革开放，发展活力明显释放。坚持问题导向，聚焦落实关键性举措，赢得发展新优势。供给侧结构性改革步伐加快。加大“三去一降一补”力度，关停搬迁东部老工业基地企业16家；济钢钢铁生产线在运行59年后实现全线停产，这是我市最大的一次产业调整，为全省去产能作出了重大贡献。认真执行国家调控政策，房地产市场基本稳定。严格落实高新技术研发费用加计扣除等财税优惠政策，降低企业“五险一金”缴纳比例，累计减轻企业负担437.9亿元。大部制改革率先实施。整合市直部门职能，逐步构建起大交通、大建设、大水务、大外事、大绿化的体制机制，打破了条块分割和城乡分治格局，为城乡一体化发展提供了强力保障。投融资改革破冰前行。整合财政资源，引导成立新旧动能转换等基金，使财政资金“四两拨千斤”的作用得到发挥；整合组建了城市投资集团、城市建设集团等六大投融资集团，将28个部门159家企业优质资产一并整合注入，国有资本资产运营能力大幅提升。人才制度改革走在全国前列。针对高层次人才短缺的问题，制定出台了“人才新政30条”“高校20条”“教育人才30条”等含金量较高的政策措施，加强长清大学城规划建设，人才环境有了明显改善；引进院士、高层次专家72人，院士（专家）工作站总数达到61个，在人才工作方面是力度最大的一年，也是成效最好的一年。全方位开放深入推进。构建开放型经济新体制综合试点试验取得积极进展，4项典型经验在全国复制推广。国家中德中小企业合作区揭牌启用，国家侨梦苑落地建设，海外孵化器、研发机构达到20多家。全市招商引资形成固定资产投资1489亿元、增长26%以上，实际到账外资125亿元、增长12%以上。成功举办市长国际经济咨询委员会首届年会、国际泉水城市联盟沙龙、中德中小企业合作大会、第十届中日韩卫生部长会等重大外事活动；国际泉水冬泳节等140余项主题节会精彩纷呈，济南“朋友圈”越来越大，城市知名度、美誉度进一步提高。

　　（六）努力保障改善民生，群众获得感明显增强。民生社会事业投入633.9亿元，占市级财政支出比重达到76%，16件为民实事全面完成。扶贫攻坚扎实推进。279个产业扶贫项目建设完工，151个贫困村完成饮水安全改造，贫困村通客车任务按期完成，1.6万贫困人口和341个贫困村实现脱贫。与湘西州、临沂市、重庆市武隆区扶贫协作取得积极进展。棚改旧改步伐加快。开工保障房68850套、建成21581套，公共租赁住房建成4744套。281个老旧小区整治改造项目全部完成，实施住宅节能改造650万平方米，惠及群众9万多户。就业取得明显成效。新增城镇就业18万人以上， 农村劳动力转移就业5.1万人，城镇登记失业率2.08%，首届全国创业就业服务展示交流活动在我市成功举办。保障能力稳步提升。连续13年提高企业退休人员养老待遇，居民养老保险基础养老金实现“七连涨”。全面实施职工和居民大病医疗保险制度，为17.9万人次减轻医疗负担4.7亿元。城镇低保标准提高到每人每月596元，农村低保、五保标准提高到每人每年4277元，慈善救助惠及困难群众16.5万人次。公立医院综合改革稳步推进，市属12家医院建立法人治理结构，完成家庭医生签约255万人。教育文化均衡发展。严格落实中小学“零择校”“零择班”，加大“大班额”问题解决力度，新建改扩建中小学187所、增加学位19万个，新建改扩建幼儿园90处、新增学位1.2万个。建立了“四点半课堂”课后服务机制，实现中小学“食堂+配餐”放心午餐模式全覆盖，更有温度的济南教育得到社会广泛好评。公共文化体系建设扎实推进，扶持新建贫困村综合文化服务中心210家，文化市场监管进一步加强。大力开展全民健身活动，实现“月月有比赛、周周有活动”。社会保持和谐稳定。大力推进城市社区党建和治理服务创新，社会组织达到3132个，注册志愿者达16万人。12345市民服务热线为群众服务633万人次，连心桥作用进一步发挥。广播电视问政促进了政风行风转变。120急救与交警指挥、应急广播联动的“生命通道”机制有效建立。全力以赴筑牢应急防控体系，圆满完成“十九大”安保维稳任务。安全生产形势总体平稳，获评“国家食品安全示范城市”，在19个副省级及以上城市的安全城市综合排名中居第三位，人民群众安全感进一步增强。史志档案、人民防空、民族宗教、红十字会和援藏援疆工作也取得了新的进步。

　　（七）切实加强政府自身建设，行政效能明显提高。认真学习贯彻十九大精神，坚持以习近平新时代中国特色社会主义思想，武装头脑，指导工作。转职能、强督查、求实效，形成雷厉风行、快干实干的浓厚氛围。工作节奏进一步加快。在治堵、治霾、拆违拆临、文明城市创建等工作任务繁重的情况下，各级各部门自我加压，迎难而上，一场硬仗接着一场硬仗打，全面或超额完成了全年目标。中建八局绿色建筑产业园等一批招商引资项目当年签约、当年开工、当年投运；市中区大涧沟、饮虎池、长清区东王等重点片区拆迁部署周密、推进有力、成效显著；顺河高架南延等一批重大基础设施建设提速、工期缩短、竣工提前，创造了“泉城速度”。工作作风进一步变实。坚持谋实事、出实招、求实效，对事关人民群众切身利益的问题，深入基层、深入群众、现场解决；对中央环保督察反馈的1399个问题，逐项核实、限时整改、对账销号；对制约济南发展的营商环境、服务环境等突出问题，注重调查研究，深入分析论证，科学制定对策措施。政务服务进一步优化。深化“放管服”改革，推出33项改革举措；工商登记实现全程电子化，一般性行政审批提速68%以上；市政府数据开放平台建成启用，实现了数据多跑路、群众少跑腿。法治意识进一步增强。认真落实人大及其常委会决定决议，依法接受人大法律监督和工作监督，主动接受政协民主监督，全年办理人大代表建议365件、政协提案691件，办结率100%。深入开展“法治六进”活动，扎实推进“七五”普法。建成全市事中事后监管平台，全面推行“双随机、一公开”监管工作，下大气力解决了一批执法不公、执法不严的问题。正风肃纪进一步严实。加大执纪审查力度，受理信访举报6406件次，立案4029件，处分3711人；查处违反中央八项规定精神问题501起，处理735人；查处侵害群众利益的不正之风和腐败问题857起，处理1056人。监察执纪工作围绕中心、服务大局，有力地保障了发展。扎实推进“两学一做”学习教育常态化制度化，把全面从严治党体现到各项工作中，党风廉政建设和反腐败斗争取得积极成效。

　　各位代表！一年来，在各级各方面共同努力下，我市很多方面发生了质的变化，干成了许多实事，解决了许多难事，迎来了许多喜事。变化有目共睹，成绩令人鼓舞。一年来的实践证明：思路清晰、方向明确，凝聚了共识，引领了发展。市委提出的“打造四个中心，建设现代泉城”中心任务，制定的“453”工作体系，契合十九大精神，符合省会实际，回应群众关切，凝聚了全市的力量，指引了发展的方向。机制顺畅、导向鲜明，激发了斗志，促进了发展。市委制定了一系列正向激励机制，为担当者担当，让实干者实惠，每年组织重点项目督查评议“三看”活动，各级各部门比着干、摽着干、放手干，形成了心齐、气顺、风正、劲足的生动局面。思想解放、敢于担当，提升了标杆，推动了发展。与好的比、向好的学，赴杭州、合肥、郑州三市学习考察，开阔了视野，转变了观念，激发了迎难而上、敢为人先的干事热情，攻克了一批城市顽疾，破解了一批发展难题。作风扎实、团结协作，形成了合力，保障了发展。几大班子精诚团结、戮力同心，各级领导带头实干、亲力亲为，广大干部爱岗敬业、履职尽责，一级带着一级干、一级做给一级看，共吹一把号、共唱一个调，形成了聚精会神抓建设、一心一意谋发展的强大合力。执政为民、凝聚人心，汇聚了力量，支撑了发展。坚持以人民为中心的发展思想，注重打造有温度的城市，广大市民始终以强烈的主人翁意识为城市发展建言献策，用辛勤的汗水为城市建设增砖添瓦。人民群众对这座城市的热爱和认同，是我们砥砺奋进、干事创业的不竭动力。

　　各位代表！去年的成绩来之不易，这是省委、省政府和市委坚强领导的结果，是市人大、市政协监督支持的结果，是全市人民团结实干、奋力拼搏的结果。在此，我代表市人民政府，向全市广大干部群众、各界人士、驻济部队、武警官兵，向中央驻济机构和省级各单位，向港澳台同胞、海外侨胞和国际友人，表示衷心的感谢和崇高的敬意！

　　在看到成绩的同时，我们也清醒认识到存在的问题和不足，主要有：城市首位度不高，省会吸引力、带动力、竞争力还不够强；实体经济实力不强，县域经济比较薄弱，开放型经济、民营经济发展还不够充分；产业层次还不够高，科技成果转化率还比较低；新旧动能转换先行区刚刚起步，携河北跨步伐亟需加快；资源环境约束趋紧，生态保护、节能减排工作需要持续用力，小清河、卧虎山水库等污染问题还比较突出；城市基础设施欠账较多，治理精细化水平需要进一步提高；民生保障存在一些薄弱环节，治霾治堵和脱贫攻坚成效仍需巩固，群众关心的就业、教育、医疗、养老等问题还没有得到很好的解决；各种不稳定不确定因素相互交织，安全生产还存在不少隐患；政府职能转变不够到位，营商环境还需优化；廉政建设有待加强，一些政府工作人员不作为、慢作为问题不容忽视，等等。对此，我们必须高度重视，勇于担当，全力加以解决。

二、2018年工作安排

　　2018年是贯彻党的十九大精神的开局之年，是改革开放40周年，是决胜全面建成小康社会、实施“十三五”规划承上启下的关键一年，也是“四个中心”建设“三年有突破”的决战决胜之年。做好新一年的工作意义重大，影响深远。

　　2018年全市经济社会发展的总体要求是：全面学习贯彻党的十九大精神，以习近平新时代中国特色社会主义思想为指导，坚持稳中求进工作总基调，坚持新发展理念，按照高质量发展的要求，以推进供给侧结构性改革为主线，以加快建设新旧动能转换先行区为契机，大力推进“1+454”工作体系，统筹做好稳增长、促改革、调结构、惠民生、防风险各项工作，推动“四个中心”建设实现“三年有突破”，让发展更有质量，改革更有成效，城市更有温度，生态更加美好，群众获得感、幸福感、安全感更加增强，加快打造“四个中心”，建设“大强美富通”的现代泉城，确保在全面建成小康社会进程中走在前列。

　　综合考虑国内外经济形势和省会发展实际，今年经济社会发展的主要预期目标是：全市生产总值增长7.5%左右；一般公共预算收入增长9%左右；固定资产投资增长12.5%左右；社会消费品零售总额增长10.5%左右；城镇居民人均可支配收入增长8%左右，农村居民人均可支配收入增长8.5%左右；城镇登记失业率控制在3.5%以内；居民消费价格涨幅控制在3%左右；单位生产总值能耗下降率及主要污染物减排量达到省控目标。今年预期目标的确定，主要考虑与“十三五”规划相衔接，坚持稳中求进，坚持高质量发展，这些目标是积极稳妥的，通过努力也是可以实现的。重点做好以下工作：

　　（一）在增强综合实力上狠下功夫，推动“四个中心”建设实现新突破。按照融会贯通、协同推进的原则，直击关键，抓住要害，推动经济发展质量变革、效率变革、动力变革，加快提升省会城市的带动力和辐射力。

　　突出实体经济，做大做强区域性经济中心。坚持质量第一、效益优先，着力构建符合省会禀赋、彰显特色优势的现代化经济体系。大力发展总部经济。坚持引培并举，进一步壮大楼宇经济。积极引进国内外大型企业在济设立总部或区域总部，引进财务公司、资管公司、科研中心、营销中心等总部型机构；加快壮大本地上市公司、大型国企总部规模，支持创新型、领军型中小企业成长为总部企业，全年新增各类总部15家，新增“亿元楼”30座以上。培育壮大新兴产业。围绕价值链打造产业链，围绕产业链部署创新链，围绕创新链配置资源链，聚焦新一代信息技术、高端装备、量子科技、生物医药、现代物流等十大千亿级产业，抓好规模扩张和提质增效，确保到2020年十大产业全部具备千亿级发展能力。大力发展数字经济，支持浪潮集团与思科、IBM等国际产业巨头合作，争创国家产业创新中心，加快山东数字经济产业园、移动智能终端产业园等建设，着力突破网络通信、高端服务器等核心关键技术，构筑国内一流的云计算和大数据产业链。积极培育先导产业，开展燃料电池大规模应用及产业化试点，推进“中国氢谷”“量子谷”等项目规划建设，力争在量子科技、集成电路、人工智能、虚拟现实、创新药物等领域实现重大突破。推动传统产业转型升级。以工业互联网、“企业上云”为抓手，促进“两化”深度融合，加快物联网、智能制造、增材制造等新技术应用，引领传统制造业加速向智能化、绿色化、低碳化迈进，让老树嫁新枝、发新芽。促进消费经济发展。加快华侨城、万达文旅城、华谊兄弟电影城、明水古城旅游度假区等项目进度，力争三年创建国家全域旅游示范区，成为国际知名旅游目的地城市。强化特色会展品牌建设，办好东亚博览会等重要展会和第十六届中国物流企业家年会。发挥宜家、奥特莱斯、华润万象城等新商圈的消费引领效应，加快布局共享经济、无人商店等新业态，培植互联网餐饮、智慧家居、品质家政等新热点，打造高端消费新高地。

　　突出产业金融，做大做强区域性金融中心。全力加快中央商务区开发建设。加速绿地山东国际金融中心等5个标志性项目建设，办好中国（济南）产业金融国际论坛，引导各类金融企业向中央商务区聚集。大力发展资本市场。加快浪潮等财务公司落地，探索设立碳排放交易市场、玫瑰资产交易中心等平台；发挥微软、甲骨文等企业引领带动作用，建立科技创新金融支持体系，吸引风险投资、产业基金、私募股权等新业态落户。深入开展企业上市推进年活动。完成规模企业规范化改制250家，建立上市企业资源数据库；强化专业服务平台建设，设立并购基金，加大对企业上市扶持力度，新增一批上市挂牌企业，全年直接融资规模突破1100亿元。加强金融风险防范。强化金融监管，完善债务风险预警机制，加大对非法集资、保险欺诈、金融信贷诈骗等违法犯罪行为打击力度，防范政府债务及企业资金链断裂等风险，保障金融安全运行。

　　突出内陆港建设，做大做强区域性物流中心。强化交通基础设施支撑。加快综合交通枢纽城市建设，研究制定城市内环、中环、外环“三环十二射”交通网络规划，全方位推进铁路、公路、航空、水运综合运输体系提级扩容，加快“米”字形高铁网建设，启动济郑、济滨、济莱等高速铁路建设，加密延长中欧班列，支持济南机场扩建，打造多式联运物流中心。强化园区载体支撑。整合提升现有物流园区，加快综合保税区迁建，扩大临空国际物流园区规模。规划建设大容量保税仓库、保税货场和集装箱中心，促进物流“大进大出”。建立物流交易信息平台，加快推进“1+6”都市圈城际配送体系建设，打造智慧物流名城。强化业态支撑。支持现有重点物流企业扩大规模，着力引进国内外知名物流企业，推进菜鸟、安博等领军物流企业区域总部尽快落地见效。积极培育无车承运和供应链体系等新业态、新模式。争取国家支持。加快申报国家一类铁路开放口岸，开辟更多国际航空货运新航线，争取设立空港保税物流中心，增强省会物流中心的吸附力和影响力。

　　突出科技成果转化，做大做强区域性科创中心。加快成果转化。依托国家科技成果转移转化示范区建设，发挥省会高校资源优势，加快建设山东工研院、意大利（济南）技术转移中心、国家人类遗传资源库山东创新中心等新型研发转化机构，培育5-10个协同创新中心，力争落地一批重大产业化项目。用好天使基金等资源，加大风险补偿，吸引社会资本助推科技成果转化，努力打造全国有影响力的科技成果交易转化高地。加大平台建设。争创国家级量子技术标准化创新基地，促进量子技术实现产业化。加快创新谷等重大创新平台基地建设，打造一批创新社区、转化社区。抓住首都高校、医疗、科研院所疏解转移机遇，招引一批大院大所。鼓励开展跨国并购，新建海外研发机构10家以上。厚植人才优势。推进济南CBD和汉峪金谷两大人力资源服务产业园建设，加快济南高层次人才创新创业基地和海外离岸创新创业基地建设，着力引进人工智能、先进材料、精准医疗等领域的顶尖创新创业团队和领军人才，新增院士工作站20家以上，引进遴选泉城引才倍增计划人才（团队）20个以上，培养支持泉城产业领军人才计划人选30个以上。加强人才梯队建设和本土人才培养，打通人才引进、使用中的体制机制障碍，打通科技和经济转移转化的通道，加大知识产权保护力度，让各类人才创造力竞相迸发，以人才优势支撑省会竞争优势。

　　（二）在积蓄后劲上狠下功夫，推动五项重点工作实现新提升。五项重点工作事关省会长远发展，必须牢牢盯住、紧抓不放，让省会发展基础更加坚实、支撑更加有力。

　　强力抓好招商引资。聚焦“专业、影响、载体、政策、环境”，提升精准招商成效。积极发展与专业中介机构的招商伙伴关系，探索实行聘任制公务员和KPI考核机制。策划组织德国济南周、香港济南周、跨国公司济南行等重大活动。依托园区开展产业链招商，引进一批龙头企业和产业项目，吸引一批配套的科技型中小微企业，推动优势主导产业集群发展。

　　强力抓好重点项目建设。发挥好有效投资对促进经济增长的关键作用，建立完善市级重点项目库，着力抓好千亿级中国重汽小镇、齐鲁航空城、中德产业园等240个市级重点建设项目，总投资超过1万亿元，完成年度投资2700亿元以上。强化土地、资金、技术等要素保障，开辟绿色通道，确保重点项目早开工、早竣工、早投产、早达效。

　　强力抓好棚改旧改。加大保障性住房供给力度。像“给父母盖房子”一样，高标准开工建设省级棚改安置房4.1万套，市级棚改安置房1.9万套。推进35个棚户区和90个城中村改造，确保到2020年全面完成棚改任务，绝不把棚户区带进小康社会。完善住房保障机制。坚持“房子是用来住的，不是用来炒的”，加快建立多主体供给、多渠道保障、租购并举的住房制度，大力发展住房租赁市场；完善促进房地产市场平稳健康发展的长效机制，保持调控政策的连续性和稳定性。继续实施老旧小区改造提升。重点启动472个老旧小区综合改造，完成整治改造749万平方米以上，积极推进既有住宅加装电梯等工作，让市民居住环境更加整洁宜居。

　　强力抓好拆违拆临、建绿透绿。坚持“一把尺子量到底”，对各类违法建设坚决拆除，大力实施“即拆、即清、即绿、即美”行动，进一步释放城市公共空间。积极推进“无违建”创建活动，严防新的违法建造产生。全面完成违法违规户外广告拆除，规范整治牌匾标识。深入开展城市杆线整治，利用两年时间清除绕城高速以内的“空中蜘蛛网”。扎实开展见缝插绿、裸土覆绿工程，提升海绵城市质量和绿化覆盖率。

　　强力抓好城市更新。围绕“城市让生活更美好”，把城市更新作为一个永久的课题来抓。提升规划水平。完成济南城市发展战略规划，开展规划期至2035年的新一轮城市总体规划修编，划定生态保护红线、永久基本农田和城镇开发边界三条控制线；推动多规合一，调整完善土地利用总体规划，完成市政设施、公共服务设施三年建设规划，构建生产美、生活美、生态美的空间格局。提升城市品质。推进“城市双修”、城市设计试点工作，全面落实历史文化名城保护规划。坚持拆、改、留并举，积极实施街景深度美化，推进“1+5”特色街区综合更新和环城带状公园建设，精心做好老商埠区等历史风貌区保护。积极推进“济南泉·城文化景观”申报世界文化景观遗产，加快明府城等泉文化核心标志区规划建设，推进“一湖一环”夜景照明工程，将“泉城夜宴”打造成为城市新靓点。加大对车站、机场等城市出入口整治力度，塑造城市门户新形象。提升智慧城市建设水平。运用智能化、大数据服务社会民生，争创国家新型智慧城市试点。推进“七通”建设，即群众办事一站通、居民健康一卡通、和谐社区一格通、爱城市网一点通、公共安全一网通、市民出行一路通、金融服务一贷通，让广大市民“手持一卡，走遍泉城”。提升城市功能。加强城市基础设施建设，加快城区天然气管网和调峰储气库建设，推进管道天然气市域全覆盖。新建旅游路等3座水厂，完成80公里老旧供水管网改造，启动870个供热自管站改造。配建教育、医疗卫生、文化体育、社区养老及超市、菜市场等民生设施，打造15分钟社区生活圈。提升创城成果。引导县和村镇争创全国文明城市和全国文明村镇，实施城市提升行动，巩固创城成果，提升城市管理和绿化标准，建立常态长效管理机制，像绣花一样管理城市，让文明成果惠及更多人民群众。

　　（三）在破除瓶颈上狠下功夫，推动四大攻坚战取得新成效。治霾、治堵、脱贫和新旧动能转换先行区建设是全市上下关注的难点和焦点，必须坚持不懈，聚力攻坚，确保取得实质性突破。

　　坚持不懈推进治霾攻坚。持续实施大气污染防治十大措施，严格落实中央环保督察要求，强化精准治霾，不靠刮风靠作风，坚决打赢蓝天保卫战。抓好清洁取暖试点城市建设。坚持集中和分布式供暖相结合，稳妥推进煤改电、煤改气工程，鼓励利用工业余热，稳步实施“外热入济”，因地制宜发展地热、生物质、太阳能等新能源供热方式。大力推行企业“蓝绿名单”，让环保信用优秀的企业安心生产，对“散乱污”企业坚决予以关闭，倒逼企业转型升级。加大治理管控力度。运用先进科技手段，对工地扬尘、道路扬尘、机动车尾气实施精准监测。对高排放老旧柴油车，实施区域限行和鼓励淘汰。做好重污染天气应急减排工作，确保空气良好以上天数持续增加，让市民群众享有更多的蓝天白云。

　　持之以恒推进治堵攻坚。标本兼治、建管并举，统筹推进高速路网、快速路网、城市主次干道、大容量公共交通、慢行系统五大城市交通体系建设，实现出行方式多元化。完善提升公共交通系统。加快R1线、R2线、R3线一期工程建设，确保R1线2019年开通运营，启动轨道交通两条M线和R3线二期工程；深化公交改革，完善运行机制，优化公交线网布局，加快主干线路大运量交通通道建设，开通工业北路、二环南路、经十东路等BRT线路，提高公交分担率。加密高速路网。加快济青高速、青兰高速、济泰高速、绕城大东环等建设，加快构筑“三环十二射”高速公路网。提升快速路网。推进北园大街快速路西延、顺河快速路南延（南外环-南绕城段）等快速路建设。优化主次干道。优化东部城区路网，完成经十一路、凤岐路、和平路东延等27条瓶颈路建设改造，提高路网密度，进一步改善交通微循环。积极运用大数据技术，促进智能交通系统建设，提高道路通行能力。完善城市慢行系统。合理分配路权，推动多种交通工具“零接驳”“零换乘”，让市民享有更多的绿色低碳出行方式。

　　精准发力推进脱贫攻坚。确保2018年底市定标准贫困人口全部脱贫。拓宽脱贫渠道。持续加大投入，实施240个产业扶贫项目，促进贫困群众就地就近就业，加强医疗、保险扶贫资金保障，全面做好脱贫兜底。巩固脱贫成果。强化已脱贫人口的跟踪扶持，稳定控制返贫发生率。完善脱贫成效评估。积极引入第三方评估，规范贫困户退出程序，确保脱贫成效经得起检验。全面实施黄河滩区脱贫迁建。加大迁建项目建设力度，力争三年内完成34.7万人安置任务，让滩区群众搬得出、稳得住、能发展、可致富。

　　举全市之力推进先行区建设攻坚。坚持以中疏支撑北跨、以北跨带动中疏，开启先行区建设“元年”，实现“千年一跨”破题起势。坚持规划先行。按照田园城市、精明增长的理念，绘制高水平“多规合一”的蓝图，同步推进总体规划与产业、交通、生态等专项规划编制，以产兴城、以城促产、以业聚人。启动“引爆区”建设。加快“三桥一隧”建设，在10平方公里范围内实施“十通一平”，鼓励优质医疗教育文化资源向黄河北岸辐射，推动一批标志性、引领性重大项目落地。打造黄河生态景观风貌带。深度挖掘黄河文化内涵，加强水系、湿地、林地等自然资源的保护利用，加快建设黄河国家湿地公园，打造黄河两岸的“绿带”和城市发展的“绿芯”。构建鹊山、华山之间的生态廊道，再现“鹊华烟雨”文化胜境。

　　（四）在激发活力上狠下功夫，推动改革开放开创新局面。今年是改革开放40周年，必须以更大的力度推进改革、扩大开放，进一步释放市场活力和社会创造力。

　　深化供给侧结构性改革。重点在破、立、降上下功夫，推动各类要素市场化配置。大力破除无效供给。对重化工等过剩产能实施腾笼换鸟，引导企业向园区整合集聚；充分发挥安全、质量、能耗、环保等领域法律法规的约束作用和技术标准的门槛作用，坚决出清“僵尸企业”。大力培育新动能。牢固树立产品思维，提升品种丰富度、品质满意度、品牌认可度，增加有效供给。开展“全国质量强市示范城市”创建，依托浪潮“五位一体”质量提升平台，构建“以质量信用企业为主体、以产品质量标准为基准、以检验检测为依托、以质量溯源为手段、以质量保险为保障”的质量管理系统，新增一批驰名商标和知名品牌，加快向质量经济、品牌经济迈进。大力降低实体经济成本。全面落实各项税收优惠政策，继续清理规范涉企收费，打好降成本“组合拳”， 让企业卸掉重负、轻装上阵。

　　深化“放管服”改革。精简一批行政审批事项，做好证照分离改革试点，完善容缺受理、“多评合一”等审批机制，进一步降低制度性交易成本。全面推行“双随机、一公开”，强化事中事后监管平台建设。加快公共服务事项网上办理，7月1日前全面实施“零跑腿”和“只跑一次”事项清单制度，确需多次办理的事项实行“你不用跑我来跑”，全力打造审批效率最高、审批事项最少、政务服务最优的“十最”营商环境。

　　深化投融资体制改革。加快由间接融资向直接融资转变等“九个转变”步伐，探索组建投融资体制改革决策委员会和专家咨询委员会，统筹全市重大投融资项目决策。抓好市属六大投融资集团整合提升，减少管理层级，聚焦主业经营，提升企业资本运作能力和市场化运作水平。发挥新旧动能转换等基金的杠杆撬动作用，提高市场化融资水平，让更多金融活水浇灌实体经济。

　　深化国企国资改革。着眼做强做优做大国有资本，加快国有企业资产证券化步伐，年内培育8家上市资源，力争3年内市属国有企业资产总额超过1万亿元。推进混合所有制改革和员工持股试点工作，出台国资监管机构权力和责任清单，建立监事会监督事项清单，提高监管效能。基本完成“三供一业”分离移交，实现国有企业“瘦身健体”；推动国有企业加快形成有效制衡的公司法人治理结构和灵活高效的市场化经营机制，修订完善业绩考核与薪酬管理办法，切实增强企业发展内生动力。

　　深化农村改革。衔接落实好第二轮土地承包到期后再承包30年的政策，探索承包地“三权”分置有效实现形式，为农民提供信贷支持。开展资源性资产确权登记颁证工作，加快经营性资产确权到户和股份合作制改革，保障农民财产权益。巩固扩大林改成果，完成11处国有林场改革任务；积极探索通过资产租赁、生产服务、联合经营等形式，加快发展壮大集体经济。

　　在深化改革的同时，进一步扩大对外开放，推动形成全方位开放新格局。在构建开放型经济新体制上持续加力。按时完成125项试点任务，在贸易和投资便利化、园区管理、体制机制创新等方面形成一批可复制可推广的经验案例。在扩大进出口规模上持续加力。积极争创国家级跨境电子商务试点城市。拓宽对外开放渠道，支持企业参与“一带一路”建设，鼓励利用第三方服务平台开拓国际市场，促进优势产能走出去。在打造国际合作平台上持续加力。推动境外经贸合作区建设，强化国际资本、品牌、技术和渠道对接，逐步形成“总部在济南，基地在海外，营销在全球”的发展运营模式。发挥国家侨梦苑集聚侨资侨智优势，吸引侨资企业落地入驻。强化对德双向交流，加快推动中德中小企业合作区建设。在深化国际交流上持续加力。办好国际泉水文化景观城市联盟年会、第二届市长国际经济咨询委员会年会等特色国际品牌活动。加快建设国际学校、国际医院、国际社区、国际文化演艺场馆等服务设施，不断强化国际化要素支撑。

　　（五）在做大做强上狠下功夫，推动县域经济实现新跨越。坚持少取多予放活、二三产业并举，着力增强县域经济综合实力。

　　大力实施乡村振兴战略。按照产业兴旺、生态宜居、乡风文明、治理有效、生活富裕的总要求，科学制定全市乡村振兴战略规划。着力发展都市农业、精致农业，坚持质量兴农、绿色兴农，积极推行一县一品、一镇一品，培植壮大章丘大葱、平阴玫瑰、商河花卉等十大特色产业，以标准化、基地化支撑农业品牌化发展。大力发展农业“新六产”，以打造15个现代农业综合体为抓手，拉长种养加产业链条，补齐农产品深加工短板；深入推进“互联网+现代农业”，积极发展乡村旅游、农村电商、休闲农业，让“工业拉着农业的手，城市带着乡村走”。着力壮大新型农业主体，推行土地入股、土地流转、土地托管等多种经营方式，实施40项农业科技创新及成果转化项目，提高农业规模化、机械化、科技化水平。全面落实粮食安全市长责任制和“菜篮子”市长负责制，确保粮食安全和蔬菜产品供应。

　　大力推进二三产业双轮驱动。实施新型工业壮大和服务业快速提升行动，着力发展适应县域的高技术、高附加值、低能耗的先进制造业。按照企业集聚、要素集约、技术集成、产业集群、服务集中的原则，加快园区整合和扩容升级，鼓励县区建设标准厂房筑巢引凤，全年建成280万平方米。推动城区产业园与县域工业园合作，共建一批大项目。落实存量工业、科研用地分割转让政策，组织开展区域环评或代评，切实降低入园企业成本。鼓励县域因地制宜发展生产性服务业和生活性服务业，打造一批特色商业街和商业综合体，推动温泉旅游、商务会展、休闲养生等产业加快发展。

　　大力推进新型城镇化。牢固树立全域规划理念，制定出台差别化扶持政策，统筹推进城乡基础设施、公共服务融合，支持济阳撤县设区，推动商河、平阴更好融入全市发展格局。坚持以产兴镇、以镇促产，引导农村人口向城镇有序转移，建设农村社区服务中心126处，创建12个小城市培育试点和特色小镇。启动农村环境综合整治三年行动计划，继续推进“七改”工程，重点打造60个省、市级美丽乡村示范村，美丽乡村标准化覆盖率提高10个百分点以上，让农村成为令人向往的地方。

　　大力发展民营经济。落实支持非公有制经济健康发展的政策措施，做好时间、商务、制度交易成本的“减法”和公共服务、生态环境、文明素质的“加法”，为企业发展创造有利环境。实施市场主体培育工程，引导中小企业向“专精特新”方向发展，培育一批单项“隐形冠军”，让“大而强”企业顶天立地，“中而优”企业遍地林立，“小而美”企业铺天盖地，力争民营经济占比达到38%以上。大力实施建名企、出名品、育名家“三名”工程，激发和保护企业家精神，鼓励更多社会主体投身创新创业。

　　大力强化资金保障。建立健全实施乡村振兴战略的财政投入保障机制，加大市区两级财权事权改革，进一步明确县域事权。将更多财政资源向县区倾斜，加大涉农资金统筹整合力度，集中切块下达到县，市区两级财政集中支持每个县区1-2个重点骨干项目。建立适合农业农村特点的农村金融体系，引导金融机构和工商资本参与乡村振兴，提供充足的资金保障。

　　（六）在生态建设上狠下功夫，推动环境治理取得新进展。坚持“绿水青山就是金山银山”的理念，努力提供更多优质生态产品，满足人民群众日益增长的优美生态环境需要。

　　以更大的力度治山。坚持“共抓大保护、不搞大开发”，健全实施生态补偿机制，持续推进南部山区生态自然景观保护，严禁新的开发建设。实施“山水林田湖草”生态保护修复工程，加大还林还草力度，完成山体绿化提升35座，开工建设山体公园20处；强化自然保护区规划建设和管理，推进云台寺、北大山等郊野公园试点建设；完成造林绿化5万亩，让人民群众在绿肺氧吧中乐享自然野趣。

　　以更大的力度治水。严格执行河长制，落实“一河一策”治理措施，全力抓好“水十条”，实施饮用水水源地环境综合整治，启动卧虎山、锦绣川水库周边村庄改造搬迁工程；开展小清河水质达标攻坚行动，完成水质净化一厂、二厂等污水处理能力扩建任务，推进城区100公里雨污分流改造，确保省控以上河流断面水质达到年度目标要求。坚持“广蓄水、储客水、保泉水”，充分利用黄河、长江等客水资源，加快白云水库、东湖水厂等供水设施建设，扩大城市供排水能力；加快水源置换，严格控采地下水，确保泉水持续喷涌，让城市更加灵动秀美。

　　以更大的力度治土。加大土壤面源污染治理，推进农用地分类分级管理。加强畜禽养殖监管，新建10万亩防治示范区；建成一批现代高效农田水利样板，完成20处高效节水灌溉示范园建设，积极推行水肥一体化，新增面积8万亩以上。坚决守住土壤环境质量底线，让土地变得更干净。

　　以更大的力度治脏。深入推进城乡环境整治，加大占道经营等治理力度，全面提升城市管理精细化水平。构建政府主导、党政机关先行、全社会广泛参与的垃圾分类收集处理体系，加快建筑垃圾资源化再利用。大力实施“厕所革命”，新建改建一批城市公厕，努力实现农村无害化卫生厕所全覆盖，补齐这块影响群众生活品质的短板。

　　（七）在办好为民实事上狠下功夫，推动民生事业取得新成果。聚焦群众需求和关切，办好18件民生实事，努力打造有温度的城市，不断增强人民群众的获得感幸福感安全感。

　　稳定扩大就业。深入实施就业优先战略，推动创业带动就业。突出做好高校毕业生、去产能企业分流职工、农村转移劳动力三大群体就业工作，落实军转干部、退役士兵安置任务。做好城镇就业困难人员和登记失业人员等帮扶工作，加强农民工服务中心和零工市场建设，抓好农民工工资清欠工作。新增城镇就业10万人，农村劳动力转移就业5万人，努力实现弱有所扶、业有所就、劳有所得。

　　拓宽社会保障。完善职工和居民医保政策，推进城乡一体化医保制度建设。提高城乡低保、农村五保补助标准，将低保和特困人员住院救助比例提高到70%以上。大力发展老龄事业，实施养老服务设施建设三年行动计划，新建县区养老中心4处、街道养老设施30处、社区老年人日间照料中心和农村幸福院177处，让更多老年人老有所养、老有所依、老有所乐。

　　发展教育事业。深入实施加快教育事业发展三年行动计划和第三期学前教育三年行动计划，完成“全面改薄”任务，新建50所幼儿园，开工建设50所中小学、幼儿园学生集中就餐场所。启动“义务教育优质均衡县区”创建工作，保障外来务工随迁子女平等入学。推动普通高中特色化、多样化发展，健全现代职业教育和培训体系，搭建高校与驻地合作平台。完善教师县管校聘改革和师德建设长效机制，稳步推进教育管办评分离，切实减轻学生课业负担，让孩子们享受到童年的快乐、学习的快乐、成长的快乐。

　　打造“健康济南”。加快济南国际医学科学中心建设，推进国家健康医疗大数据中心、齐鲁医科大学等项目落地动工。深入实施医疗卫生服务体系规划，加快市传染病医院、市中医院东院区、市精神卫生中心建设，新建社区卫生服务机构39所、改扩建16所；深化医药卫生体制改革，完善分级诊疗制度，全面推进家庭医生签约服务；加大妇幼健康服务资源供给，提升优生优育水平。加快健康城市、健康村镇建设试点和省级医养结合示范市建设，促进健康事业、健康产业两翼互动。实施食品药品安全六大工程，食品抽检达到每千人7份，保障广大市民舌尖上的安全。广泛开展群众性体育活动，大力发展体育事业和体育产业，积极申办国际性、全国性大型体育赛事，策划制造更多的兴奋点，让城市充满生机、充满朝气、充满活力。

　　发展文化事业。推进公共文化体系建设，发掘弘扬泉水文化、名士文化等特色文化，持续开展“书香泉城”全民阅读活动，精心策划一批具有齐风鲁韵的文化演艺活动，擦亮城市文化品牌。积极创建国家文化消费试点城市，打造国家传统工艺工作站，筹备举办第五届中国非遗博览会，承办第七届山东文博会；积极培育一批有全国影响力的文化交易市场，做大做强影视传媒、创意设计、数字资讯等优势产业。加快省自然博物馆、市博物馆新馆、市档案馆新馆、市方志馆、泉文化博物馆建设。开展纪念济南解放70周年系列活动，高水平完成济南战役纪念馆布展，加大革命遗址保护力度。实施文物拯救行动，加快城子崖、华阳宫等重点文化遗产项目保护建设，打造齐长城人文自然风景带，让人民群众寻得到文脉、记得住乡愁。

加强社会治理。积极做好信访、仲裁、人民调解等工作，妥善化解房地产等领域的社会矛盾。完成村和城市社区“两委”换届选举工作，配齐配强“两委”班子。深化平安济南建设，统筹推进“雪亮工程”，打造让人民群众放心安心的安全城市。精准铁腕整治安全隐患，确保安全生产形势持续稳定。加强互联网安全管理，构建清朗网络空间。深化双拥共建，扎实推进国防动员准备，促进军民融合深度发展。支持工会、共青团、妇联、红十字会等人民团体广泛参与社会治理，扎实做好哲学社会科学、防震减灾等工作，全面推动各项工作上档次、上水平、上台阶。

三、坚持以人民为中心，努力建设人民满意政府

　　打铁必须自身硬。全市政府系统必须坚持和加强党的全面领导，牢固树立“四个意识”，始终绷紧讲政治这根弦，进一步强化雷厉风行、快干实干的作风特色，建设为民、务实、清廉政府。

　　（一）深入推进责任政府建设。以造福人民为最大政绩，想群众之所想，急群众之所急，把工作做到老百姓惦念和揪心的地方。大兴调查研究之风，广泛征求群众意见，进一步发挥12345市民服务热线的作用，让政府决策过程成为汇聚民意、集中民智、凝聚民心的过程。按照政治过硬、本领高强的要求，加强公务员队伍建设，培养专业能力，弘扬专业精神，做到凭专业说话，按程序办事。既注重雷厉风行，更注重久久为功，一件一件抓落实，一步一个脚印，脚踏实地干好工作。幸福都是奋斗出来的，天上不会掉馅饼。要始终同人民群众想在一起、干在一起，真正担负起兴一方经济、富一方百姓、建一方文明、保一方稳定的政治责任。

　　（二）深入推进法治政府建设。认真落实法治政府建设实施纲要，切实做到法无授权不可为、法定职责必须为。依法接受人大及其常委会法律监督和工作监督，自觉接受人民政协民主监督，主动接受社会和舆论监督，认真听取人大代表、政协委员、民主党派、工商联、无党派人士和人民团体的意见。深入推进跨部门综合执法和执法力量向基层下沉，做好政府立法工作，加强行政复议规范化建设，做好行政应诉工作。加强审计工作，强化政务公开，规范公共资源交易和政府采购，让权力在阳光下运行，提高政府公信力。

　　（三）深入推进高效政府建设。落实容错纠错、正向激励机制，鼓励广大干部干事创业。树立营商环境就是生产力、竞争力，必须坚持不懈地优化发展环境的观念。带着责任和感情当好企业服务员，做到“有求必应、无事不扰”。加快“红顶中介”与行政机关脱钩，严厉整治各类“黑中介”，对吃拿卡要、办事不公等问题重拳出击，坚决清除奇葩证明等乱象。深入推进信用体系建设，建立失信联合惩戒机制，让守信者扬眉吐气、失信者名誉扫地，打造诚信济南。

　　（四）深入推进廉洁政府建设。坚持把纪律和规矩挺在前面，认真贯彻中央八项规定及实施细则精神，继续整治“四风”问题，坚决反对特权思想和特权现象。扎实开展“不忘初心、牢记使命”主题教育活动，把“忠诚”“看齐”写在岗位上，刻在事业中。加大扶贫领域腐败和作风突出问题专项治理，严肃查处侵害群众利益行为。保持惩治腐败高压态势，做到无禁区、全覆盖、零容忍，努力实现干部清正、政府清廉、政治清明，推动形成正气充盈的政治生态。

　　各位代表，乘风好破浪，奋进正当时！省会的发展已站在新的历史起点上，让我们紧密团结在以习近平同志为核心的党中央周围，在省委省政府和市委的坚强领导下，不忘初心、牢记使命，雷厉风行、快干实干，为加快“打造四个中心，建设现代泉城”而努力奋斗！
